# Supplementary material for: Splice-Junction-Based Mapping of Alternative Isoforms in the Human Proteome
Source: Cell Rep. Author manuscript; Available in PMC 2020 Jan 15. (PMC6961840; doi:10.1016/j.celrep.2019.11.026)
Supplement: 2 [file NIHMS1546469-supplement-2.zip › DF1/PXD010154/AdrenalGland/AdrenalGland_16_RAP1GAP_WLPPAPLPRPLSITPPR.pdf]

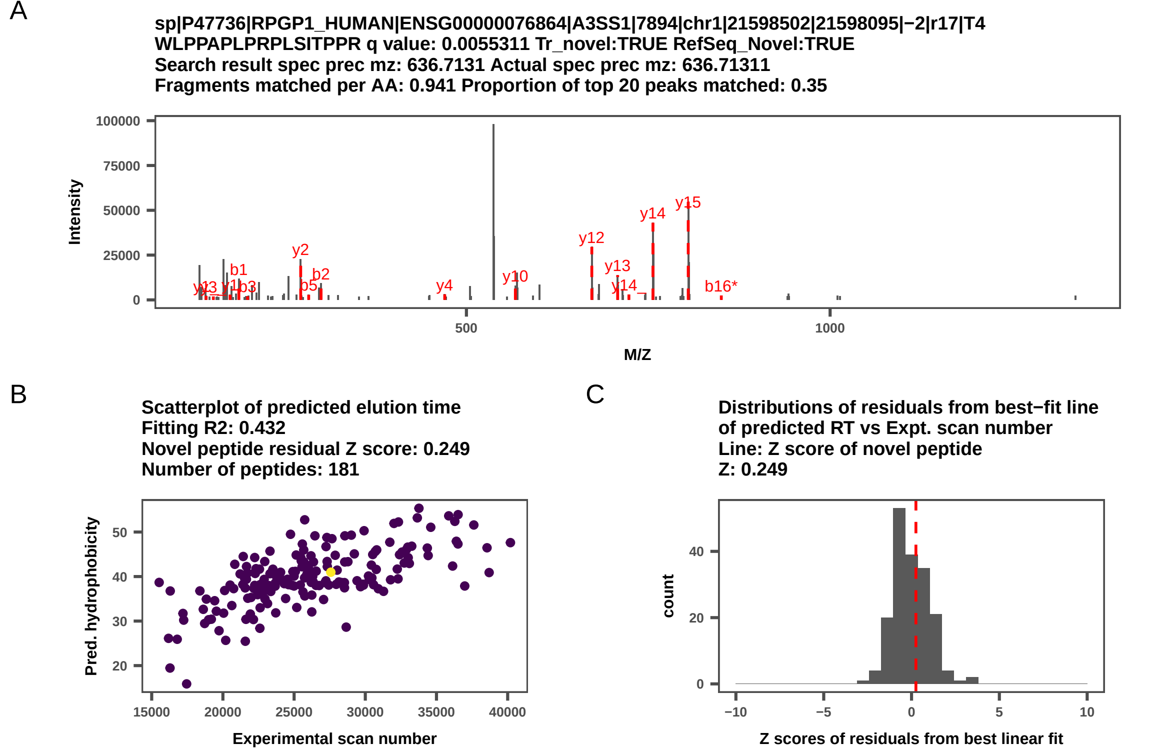
A
sp|P47736|RPGP1_HUMAN|ENSG00000076864|A3SS1|7894|chr1|21598502|21598095|−2|r17|T4
WLPPAPLPRPLSITPPR q value: 0.0055311 Tr_novel:TRUE RefSeq_Novel:TRUE
Search result spec prec mz: 636.7131 Actual spec prec mz: 636.71311
Fragments matched per AA: 0.941 Proportion of top 20 peaks matched: 0.35
Intensity
100000
75000
50000
25000
0
y15
y14
y12
y13
y2
b1
b2
y10
y4
b5
y14
b16*
500
1000
M/Z
B
Scatterplot of predicted elution time
Fitting R2: 0.432
Novel peptide residual Z score: 0.249
Number of peptides: 181
Pred. hydrophobicity
50
40
30
20
15000
20000
25000
30000
35000
40000
Experimental scan number
C
Distributions of residuals from best−fit line
of predicted RT vs Expt. scan number
Line: Z score of novel peptide
Z: 0.249
count
40
20
0
−10
−5
0
5
10
Z scores of residuals from best linear fit
